# RET fusions observed in lung and colorectal cancers are sensitive to ponatinib

**DOI:** 10.18632/oncotarget.25664

**Published:** 2018-07-03

**Authors:** Joseph M. Gozgit, Tzu-Hsiu Chen, Youngchul Song, Scott Wardwell, Frank Wang, Jie Cai, Henry Li, Henrik Edgren, Victor M. Rivera, Justin Pritchard

**Affiliations:** ^1^ ARIAD Pharmaceuticals, Inc., a wholly owned subsidiary of Takeda Pharmaceutical Company Ltd, Cambridge, MA, USA; ^2^ Crown Bioscience, Shanghai, P.R. China; ^3^ Medisapiens Ltd., Helsinki, Finland; ^4^ Department of Biomedical Engineering, The Pennsylvania State University, State College, PA, USA

**Keywords:** RET, colorectal cancer, non-small cell lung cancer, ponatinib, tyrosine kinase inhibitor

## Abstract

Genomic studies are revolutionizing clinical oncology, but bridging the lab and the bedside requires the ability to efficiently interrogate rare genetic lesions in unexpected pathological settings using preclinical models. Oncogenes can exhibit intrinsic drug resistance to targeted therapy in different cells of origin, adding complexity to clinical interpretations of genomic findings. Here, we capitalize on the flexibility of engineered cell systems to rapidly profile known multi-kinase inhibitors that harbor rearranged during transfection (RET) kinase activity across multiple RET fusions. Identifying ponatinib as the most potent RET inhibitor tested, we used ponatinib to gauge therapeutic responsiveness in RET fusion-positive patient-derived xenograft (PDX) models. Using a genomics guided outlier approach, we identified 4 RET fusion PDX models with 3 different fusion partners (KIF5B, CCDC6, and NCOA4) in both non-small cell lung cancer and colorectal cancer. By comparing ponatinib activity in RET fusion-positive and RET fusion-negative PDX models alongside a standard of care chemotherapeutic agent, we show that RET fusions in colorectal tumors are therapeutically responsive to RET inhibition. Finally, we suggest that coupling engineered cell systems and genomics guided PDX model selection provides a rapid workflow to triage rare genomics findings.

## INTRODUCTION

Genomic medicine has led to a paradigm shift in clinical trials of cancer treatments, as large-scale sequencing of relapse/refractory patients often identify rare genetic alterations in novel tumor pathologies [1]. For instance, the identification of the *BRAF V600E* mutation in a subset of colorectal cancers (CRC) created much enthusiasm for treatment with vemurafenib, but subsequent studies showed that vemurafenib is not active in CRC because of the presence of a growth factor mediated feedback loop [2]. Thus, a different tissue type provides a distinct cellular context that can create drug resistance in even a canonically druggable driver gene mutation. Therefore, genomic medicine requires preclinical studies that investigate molecular and pathological diversity in relevant preclinical models regardless of tissues of origin.

The rearranged during transfection (RET) receptor tyrosine kinase is activated in several cancers by somatic mutations or chromosomal rearrangements. While *RET* mutations have been associated with thyroid cancer for many years [3], the discovery of *KIF5B-RET* fusions in non-small cell lung cancer (NSCLC) [4] has created particular excitement. The excitement partially stems from the fact that anaplastic lymphoma kinase (ALK) inhibitors have had a profound impact on the treatment of patients with NSCLC harboring ALK fusions [5]. Thus, many hope that RET inhibitors might have a similar impact on treatment of NSCLC patients with RET fusions. Beyond lung cancer, RET fusions with a diversity of fusion partners (KIF5B, NCOA4, CCDC6, BCR, GOLGA5) have been identified in spitzoid neoplasms, chronic myelomonocytic leukemia and CRC [3]. Given this, we propose that prioritization of rare genomic events (such as novel fusion partners) in diverse indications can be achieved by coupling rapid engineered cell models with physiologically relevant PDX models.

The multi-targeted tyrosine kinase inhibitors (TKIs) vandetanib and cabozantinib harbor serendipitous RET activity, and have been approved by the United States Food and Drug Administration for the treatment of locally advanced and metastatic medullary thyroid cancer [3]. In NSCLC, several case reports showing clinical activity in RET fusion-positive patients treated with either vandetanib [6, 7] or cabozantinib [8, 9] suggested that RET fusions may be a driver in this cancer type. But further clinical trials in more patients have shown that response rates with these and other multi-targeted TKIs (e.g. sunitinib, sorafenib, and lenvatinib) only range from 18% to 37% [10]. This suggested that RET fusions do not display ALK-fusion like sensitivity in NSCLC with currently tested inhibitors.

Ponatinib is a multi-targeted TKI with potent activity against native BCR-ABL and a broad range of mutants including its gatekeeper mutation T315I, and is approved for patients with refractory Philadelphia-positive leukemias [11]. In addition to BCR-ABL, ponatinib inhibits a number of other kinases involved in cancer, including RET (and others such as KIT, fibroblast growth factor receptor, and vascular endothelial growth factor receptor) at low-nanomolar concentrations [12]. Ponatinib potently inhibits activating variants of RET in models of thyroid cancer [13, 14], and others have shown that ponatinib inhibits KIF5B-RET fusions in genetically engineered models (but not patient-derived xenograft [PDX] models) [15].

We present data across multiple fusion partners and PDX models from 2 pathological indications to show that ponatinib is the most potent of the 6 RET TKIs tested. We also confirm that RET fusion-positive CRCs are sensitive to RET inhibition. Moreover, we suggest that our work constitutes a flexible and rapid workflow for genomic medicine: 1) characterize multiple rare molecular events in a simple to use engineered cell system; 2) mine PDX genomic databases for evidence of tumors harboring those events; 3) compare PDX responses in tumors harboring a relevant event with those that do not to demonstrate genetic specificity. We believe that these steps are critical in coupling genomic and preclinical findings.

## RESULTS

### Ponatinib potently inhibits RET fusions in *in vitro* engineered Ba/F3 cell assays

**Figure 1:**
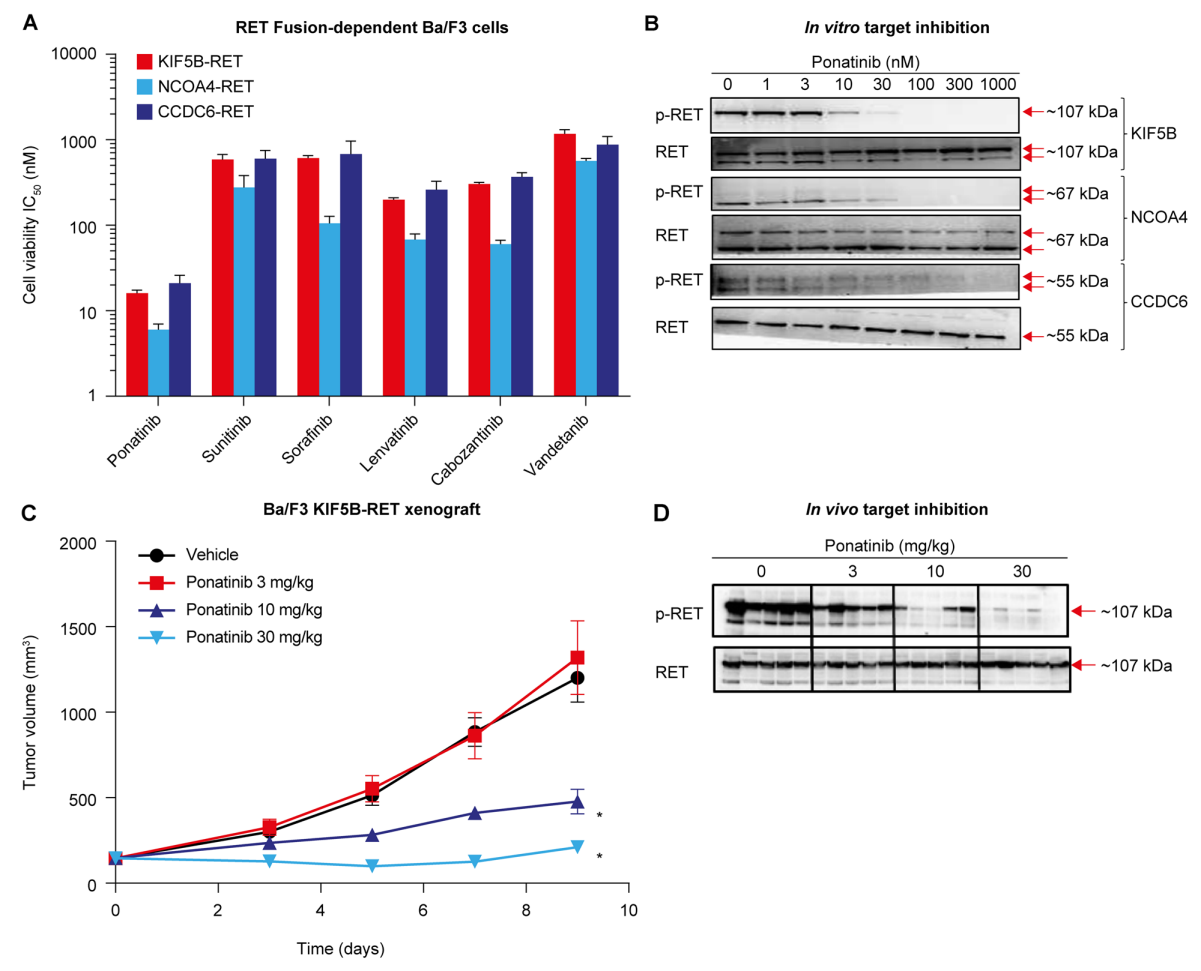
Ponatinib potently inhibits RET fusions in *in vitro* and *in vivo* Ba/F3 models **(A)** IC_50_ values (nM) of ponatinib, vandetanib, cabozantinib, sunitinib, sorafenib, and lenvatinib in Ba/F3 cells harboring KIF5B-RET, NCOA4-RET, or CCDC6-RET fusion proteins are shown. The cell lines were treated with increasing concentrations of drug for 3 days followed by cell viability assessment. Data are shown as mean ± SD from 3 separate experiments. **(B)** RET-fusion engineered Ba/F3 cells were treated with ponatinib for 1 hour, lysates were immunoblotted for phosphorylated RET (p-RET) and then reprobed for RET. Results presented are representative of 2 independent experiments. **(C)** *In vivo* efficacy of ponatinib in subcutaneous tumor model using Ba/F3 KIF5B-RET cells. Tumor-bearing animals were treated once-daily by oral gavage with vehicle or the indicated doses of ponatinib for 9 days. Mean tumor volume and SEM are plotted. Each treatment group was compared with the vehicle group using 1-way ANOVA (^*^P < 0.05). **(D)** Ponatinib-mediated pharmacodynamic effect observed in Ba/F3 KIF5B-RET tumor models. Mice were administered a single oral dose of vehicle or ponatinib at 3, 10, or 30 mg/kg and tumors were collected 6 hours later. Each lane represents a separate animal.

To compare ponatinib’s activity against RET fusions with those of other TKIs with known anti-RET activity (sunitinib, sorafenib, lenvatinib, cabozantinib, and vandetanib), we engineered Ba/F3 cell lines whose viability was RET fusion-dependent. Using this model, we evaluated the activity of each TKI against 3 distinct RET fusions (KIF5B-RET, NCOA4-RET, and CCDC6-RET, N=3 per drug). Sunitinib, sorafenib, lenvatinib, and cabozantinib displayed relatively similar anti-RET activity (IC_50_s within 2- to 5-fold) for each fusion (Figure 1A and Table 1), and vandetanib exhibited the weakest anti-RET activity against these fusions (IC_50_ 565-1172 nM). Compared with the other TKIs tested, ponatinib displayed the most potent inhibition of KIF5B-RET, NCOA4-RET, and CCDC6-RET (IC_50_ 16, 6, and 21 nM, respectively). Anti-RET activity of ponatinib was ∼12-46-fold enhanced in comparison with that of sunitinib, sorafenib, and lenvatinib, and ∼10-19-fold and ∼46-94-fold enhanced compared with that of cabozantinib and vandetanib, respectively, across the 3 fusions tested. Ponatinib was ∼100-fold more potent when tested in engineered RET fusion-dependent Ba/F3 cells versus the parental Ba/F3 line (Table 1). We confirmed that ponatinib-mediated potent inhibition of RET fusion-driven Ba/F3 cell viability was associated with corresponding suppression of RET^Tyr905^ phosphorylation (p-RET) with IC_50_s of 9, 13, and 36 nM for KIF5B-RET, NCOA4-RET, and CCDC6-RET, respectively (Figure 1B).

**Table 1:** Summary of IC_50_ viability values in RET fusion-dependent Ba/F3 cell lines

	IC_50_ ± SD, nM			
	KIF5B-RET	NCOA4-RET	CCDC6-RET	Parental
Ponatinib	16 ± 1	6 ± 1	21 ± 5	1021 ± 177
Vandetanib	1172 ± 143	565 ± 40	874 ±219	7401 ± 2633
Cabozantinib	303 ±14	60 ± 7	366 ± 46	5697 ±1134
Sunitinib	588 ± 87	277 ± 105	602 ± 147	3422 ± 783
Sorafenib	609 ± 42	105 ± 22	678 ± 286	6771 ± 2321
Lenvatinib	199 ± 11	68 ± 11	260 ± 68	>10,000

IC_50_, the half-maximum inhibitory concentration; RET, rearranged during transfection; SD, standard deviation.

Encouraged by ponatinib’s *in vitro* activity against RET fusions, we examined the antitumor effects of ponatinib *in vivo* in mice with subcutaneous tumors of KIF5B-RET-dependent Ba/F3 cells (N=10 per treatment condition). Once-daily oral administration of ponatinib resulted in a dose-dependent inhibition of tumor growth, with 30 mg/kg inducing nearly complete tumor stasis (Figure 1C). Consistent with the dose-dependent anti-tumor activity of ponatinib, corresponding inhibition of p-RET in tumors *in vivo* was also observed 6 hours post treatment (Figure 1D). Additional evidence of the specificity of ponatinib’s antitumor activity was observed when ponatinib had minimal effects on an *in vivo* isogenic Ba/F3 tumor model dependent on oncogenic EML4-ALK (Supplementary Figure 1) in comparison with the approved ALK inhibitor crizotinib (200 mg/kg). Together, these data demonstrate that ponatinib potently inhibits the NSCLC-related KIF5B-RET fusion in mice, in a target-specific manner. Collectively, these findings highlight ponatinib’s superior activity against NSCLC RET fusions tested (KIF5B-RET, NCOA4-RET, and CCDC6-RET) in comparison with a broad range of TKIs, including those that are currently being most actively evaluated in NSCLC (cabozantinib and vandetanib).

### Ponatinib shows anti-tumor activity in a clinically relevant NSCLC PDX model

**Figure 2:**
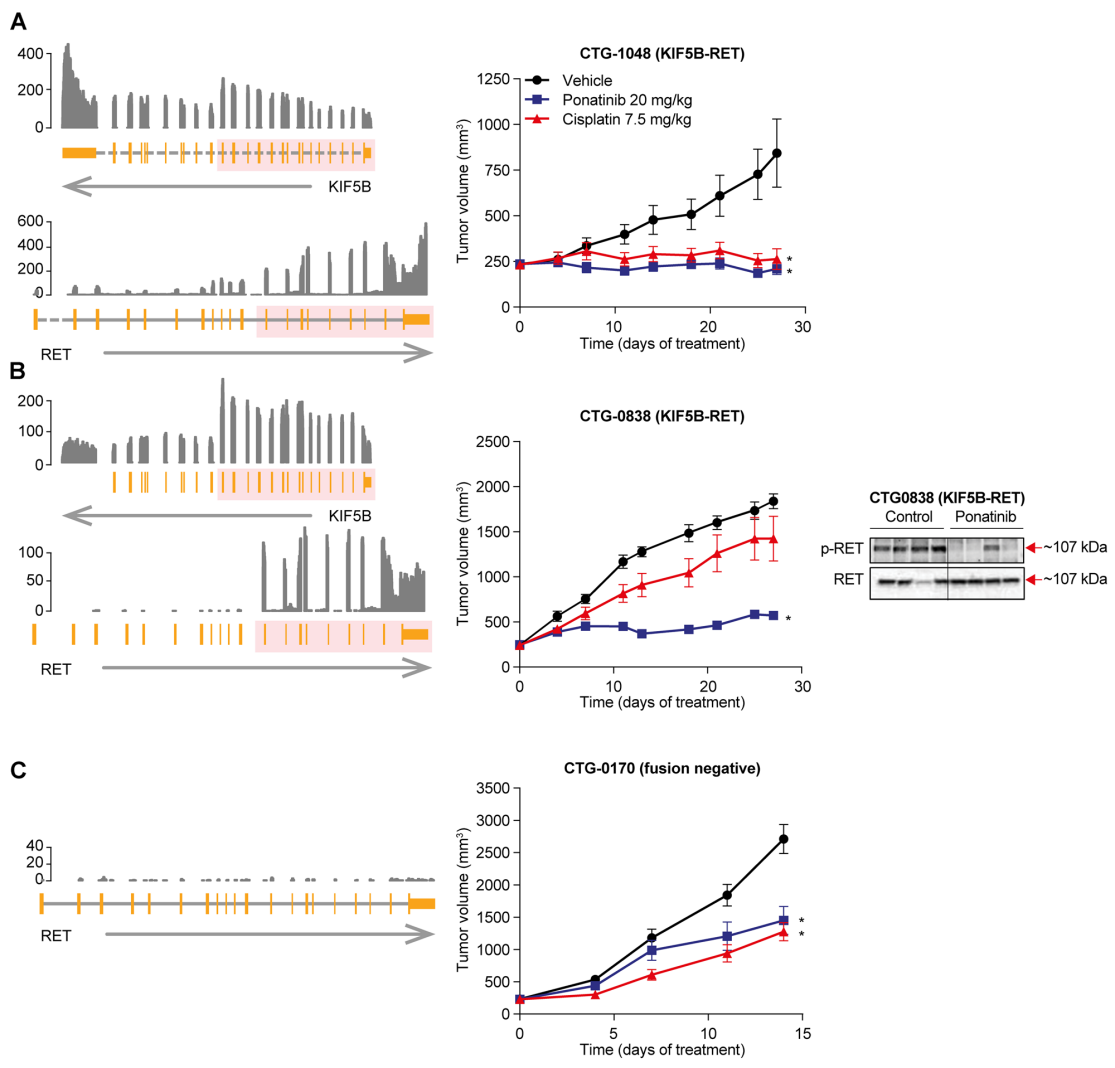
Anti-tumor activity of ponatinib in KIF5B-RET NSCLC PDX models Left panel, Schematic representation of the KIF5B-RET rearrangement in RET-fusion positive NSCLC tumor models CTG-1048 **(A)**, CTG-0838 **(B)**, and RET fusion negative CTG-0170 **(C)**. From the bottom, an arrow shows the strand of each gene, with the gene structure drawn above. Dashed lines indicate introns not drawn to scale. The pink overlay shows the exons taking part in the fusion. Normalized RNA sequencing coverage is drawn above. Only RET exons included in the fusion are expressed. Middle panel, Mice bearing patient-derived NSCLC tumor models were evaluated for ponatinib or cisplatin sensitivity. Ponatinib (20 mg/kg q.d. orally) and cisplatin (7.5 mg/kg once-weekly i.p.) were administered for 28 days. Mean tumor volume and SEM are plotted. Each treatment group was compared with the vehicle group using 1-way ANOVA (^*^P < 0.05). Right Panel, Pharmacodynamic effect of ponatinib treatment in KIF5B-RET NSCLC PDX model CTG-0838, was assessed. Mice were administered a single oral dose of vehicle or ponatinib (20 mg/kg) and tumors were collected 6 hours later. Each lane represents a separate animal.

To test ponatinib’s ability to inhibit RET fusions in a more clinically relevant context, we assessed 2 KIF5B-RET-positive NSCLC PDX models. Historically, the standard of care in patients with newly diagnosed NSCLC is platinum doublet chemotherapy, but the response rate (∼30%) and progression-free survival (PFS; 5-6 months) are low [16, 17]. To examine inhibition of RET in the context of both treatment naïve and chemotherapy-refractory tumors, we turned to 2 KIF5B-RET NSCLC PDX models (N=10 replicates/condition). Representing the frontline setting, CTG-1048 model was established from a tumor that was resected from a treatment-naïve patient with NSCLC adenocarcinoma. Consistent with being a treatment-naïve tumor, we observed 95% tumor growth inhibition, but no tumor regression in response to cisplatin (7.5 mg/kg, intraperitoneal injection [i.p.], once weekly) in CTG-1048. However, once-daily oral dosing of ponatinib (20 mg/kg) was able to induce rapid tumor regression in CTG-1048 tumors (11.2%) (Figure 2A; Supplementary Table 1). Next, we tested a second xenograft model, CTG-0838, which was established from a chemotherapy-refractory tumor. Consistent with this tumor’s chemo-refractory history, CTG-0838 tumors were considerably less sensitive to treatment with chemotherapy (cisplatin 7.5 mg/kg, tumor growth inhibition of 26%); however, ponatinib (20 mg/kg) showed significant tumor inhibition (80%) (Figure 2B; Supplementary Table 1). It is important to note that the *in vivo* antitumor activity of ponatinib was associated with inhibition of p-RET in both PDX models (Figure 2; Supplementary Figure 2).

To evaluate the specificity of ponatinib’s antitumor activity for RET, we also tested ponatinib in a NSCLC PDX model, established from a treatment-naïve patient that did not contain a RET fusion (as determined by RNA-seq) (Figure 2C). Ponatinib showed moderate tumor growth inhibition (51%), which was modestly lower than that of cisplatin (tumor growth inhibition of 58%). This indicates that ponatinib’s superior antitumor effects when compared with chemotherapy in the NSCLC PDX models are RET fusion specific.

Ponatinib treatment was well tolerated in mice, whereas 4 drug-related deaths and moderate but significantly more body weight loss was observed in the cisplatin-treated mice (*P* = 0.0037, by Wilcoxon paired test of mean percent body weight loss across all days, *N* = 20) (Supplementary Figure 3). Taken together, this suggests that the anti-tumor activity of a tolerable dose of ponatinib meets or exceeds that of a less tolerable dose of cisplatin in both chemotherapy refractory and chemotherapy naïve NSCLC PDX models.

### An outlier analysis identifies RET fusions in CRC PDX models

**Figure 3:**
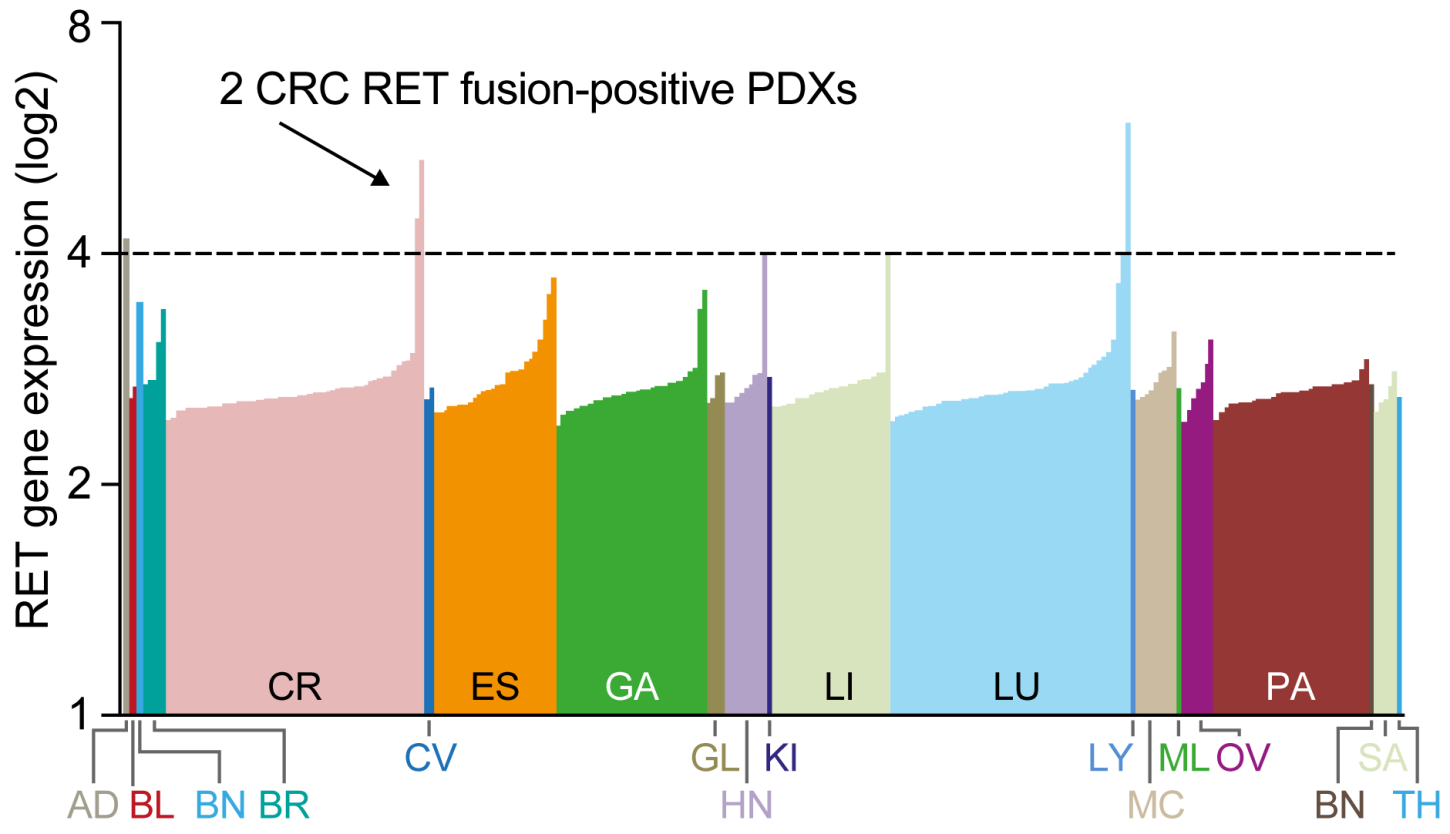
Identification of RET rearrangements in 2 CRC models Expression of RET in 273 patient-derived xenograft (PDX) samples across 20 tumor types determined by RNAseq.

**Figure 4:**
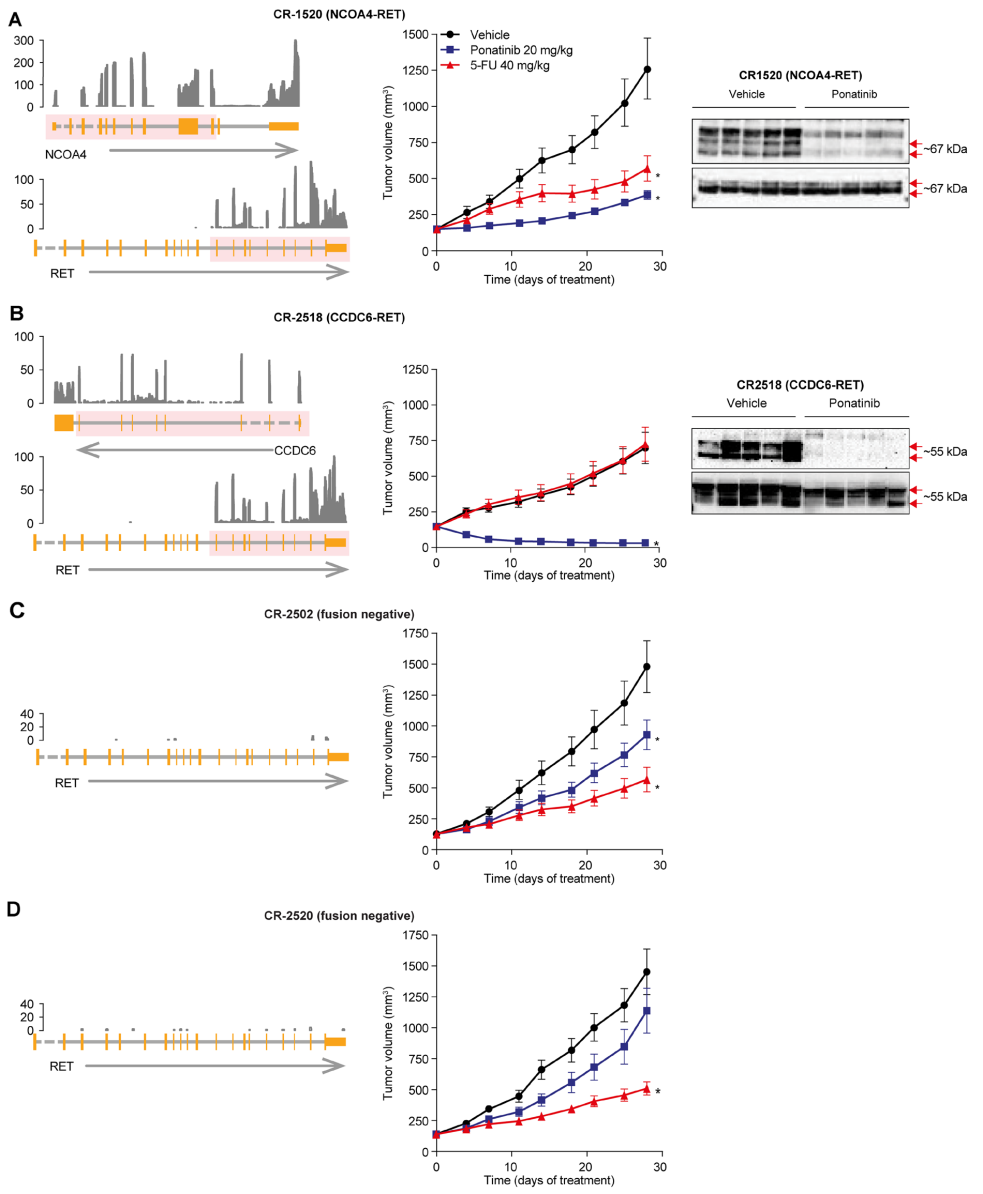
Anti-tumor activity of ponatinib in CRC PDX models *In vivo* efficacy of ponatinib in RET fusion-positive (CR1520 **(A)** and CR2518 **(B)**) and -negative (CR2502 **(C)** and CR2520 **(D)**) CRC PDX models. Left panel, Schematic representation of the RET rearrangements: NCOA4-RET (CR1520, top) and CCDC6-RET (CR2518, bottom) fusions as well as the samples with no fusions, which were identified using RNA-seq. From the bottom, an arrow shows the strand of each gene, with the gene structure drawn above. Dashed lines indicate introns not drawn to scale. The pink overlay shows the exons taking part in the fusion. Normalized RNA sequencing coverage is drawn above. Only RET exons included in the fusion are expressed. Middle panel, Ponatinib (20 mg/kg q.d. oral dosing) or 5-FU (40 mg/kg, twice-weekly i.p.) were administered for 28 days to mice bearing patient-derived CRC tumors. Mean tumor volume and SEM are plotted. Statistical significance was calculated using 1-way ANOVA (^*^P < 0.05) in which each treatment group was compared with its vehicle. Right panel, Pharmacodynamic effect of ponatinib treatment in RET fusion-positive CR1520 and CR2518. Each lane represents a separate animal.

To understand the oncogenic role of RET fusions in other cancer types, we screened the transcriptomes of a diverse set of 273 PDX samples from 20 different tumor types for *RET* fusions using RNA-seq. Most PDXs showed relatively low expression of RET; however, 2 separate CRC PDX samples and 1 NSCLC sample showed outlier RET expression (Figure 3). Despite the high RET expression observed in the NSCLC model, no RET fusion was detected. However, the transcriptome data from the CRC PDX samples revealed a noticeable exon imbalance in both CRC samples with exons 12-20 exclusively expressed (Figure 4A, 4B left panel), suggesting that RET expression in these tumors is the result of a chromosomal rearrangement. Follow-on analyses of split and spanning reads suggested that in model CR1520 *RET* is fused to *NCOA4* exons 1-10, whereas in model CR2518 *RET* is fused to *CCDC6* exons 1-8. Importantly, both RET fusion-positive tumors were negative for other major hotspot mutations, including KRAS, BRAF, and PI3K (not shown), suggesting that RET may be primary oncogenic driver in these tumors.

To validate the fusion gene breakpoint, cDNA was harvested from independent tumor samples and amplified by polymerase chain reaction (PCR). Sanger sequencing confirmed both fusions in models CR1520 and CR2518 (Supplementary Figure 4). In contrast, no PCR product was amplified from 2 CRC models, CR2502 and CR2520, which expressed relatively low RET mRNA levels suggesting that these models are negative for the respective fusions.

### Efficacy of ponatinib in CRC PDX models

We next took a similar approach as we did for our NSCLC xenografts to evaluate our PDX models of colorectal cancer. Mice with subcutaneous PDX tumors were dosed with ponatinib (N=10 replicates per condition, 20 mg/kg orally once daily) or the standard of care agent 5-flourouracil (5-FU) (N=10 replicates per condition, 40 mg/kg i.p., twice weekly). Ponatinib displayed 79% tumor growth inhibition in the NCOA4-RET model (CR1520) and near complete regression in the CCDC6-RET model (CR2518; Figure 4A, 4B; Supplementary Table 1). In both cases, ponatinib’s efficacy was greater than 5-FU. Ponatinib’s efficacy in these RET fusion-positive PDXs was also associated with the expected pharmacodynamic suppression of RET phosphorylation (Figure 4A, 4B).

To further evaluate the RET specificity of ponatinib’s antitumor activity, we tested ponatinib in the RET fusion-negative PDX models CR2502 and CR2520 (N=10, Figure 4C, D). Ponatinib’s antitumor effects were substantially decreased in these RET fusion-negative models (Figure 4C, D; Supplementary Table 1). In comparison with the RET fusion-positive PDXs, RET fusion-negative tumors were relatively more responsive to the standard of care chemotherapeutic 5-FU than they were to ponatinib (Figure 4A–4D, Supplementary Table 1). In summary, we identified recurrent RET fusions in CRC and we validated ponatinib’s ability to target these fusions in a RET-specific manner that meets or exceeds a standard of care chemotherapy agent in clinically relevant *in vivo* models.

While no deaths related to 5-FU occurred in our treatment arm, ponatinib induced significantly less body weight loss than 5-FU (*P* = 0.0086, by Wilcoxon paired test of mean percent body weight loss across all days, *N* = 26) (Supplementary Figure 5).

## DISCUSSION

The diagnosis of ALK fusions in NSCLC has led to the development of potent and selective ALK inhibitors that have dramatically changed the treatment landscape for patients who present with late stage metastatic disease [18]. The efficacy of ALK inhibitors has been so profound that even second-line treatment with a next generation ALK inhibitor like brigatinib can produce a median PFS of more than 1 year [19]. The discovery of RET fusions in NSCLC has fueled similar excitement, but the clinical and biological data are still evolving. Following the discovery of RET fusions in NSCLC there were several case reports showing clinical activity of compounds with RET activity [6–9]. These successes spurred a steady stream of clinical trials with multi-targeted TKIs that serendipitously harbor RET activity. However, it is still unclear whether RET fusion-positive NSCLC patients can achieve responses that are as impressive as ALK fusion-positive NSCLC patients dosed with ALK inhibitors. In addition to its discovery in NSCLC, RET fusions have also been identified in CRC [20]. While rare, these fusions exhibit many of the same genomic hallmarks as RET fusions in NSCLC.

We characterized ponatinib’s anti-RET activity using an engineered *in vitro* system to measure drug potency. We showed that ponatinib inhibits RET fusions *in vitro* with potencies substantially exceeding those of other RET inhibitors currently in clinical trials. Furthermore, we show this across 3 different fusion partners (KIF5B, NCOA4, and CCDC6). Testing fusion partner diversity is important to gain confidence for clinical testing and is not typically performed [15]. It is tempting to assume that all fusion partners behave the same, but validating this assumption adds confidence in utilizing RET inhibitors in basket trials where genomics-guided fusion searches can turn up a variety of lesions with diverse fusion partners in very rare patient populations.

Finally, we used clinically relevant PDX models to demonstrate efficacy. Importantly, we compare ponatinib activity in PDX models that are RET fusion-positive and RET fusion-negative. This comparison supports the hypothesis that ponatinib’s effect is dependent upon the RET fusion in NSCLC and CRC. Across both indications ponatinib had substantial activity in our RET fusion models, but not in models that lacked a RET fusion. While ponatinib has multiple targets, it represents the most potent RET inhibitor to be used in PDX models to date. We suggest that the data in these PDX models make a compelling case for further clinical investigation of ponatinib in RET fusion-positive malignancies.

Colorectal malignancies have been a difficult indication for targeted therapies. The discovery of *BRAF* alterations in CRC excited the field with the potential to repurpose vemurafenib from melanoma to CRC [21]. However, the biology turned out to be more difficult in CRC with feedback through growth factor signaling limiting responses to vemurafenib [2]. Thus, the preclinical validation of even well described oncogenes like *BRAF* is necessary when switching clinical indications. Our work independently confirms the presence of RET fusions in CRC and supports the notion of recurring oncogenic RET fusions in CRCs. Furthermore, we are the first to show ponatinib’s activity in RET fusion-positive CRC.

Our paper adds significantly to the previous literature [15, 22]. Importantly, we include multiple PDX models across both NSCLC and CRC, and we also include important controls that were omitted from other published studies by comparing efficacy in RET fusion-positive tumors to RET fusion-negative tumors, and RET kinase inhibition to standard of care chemotherapy. Moreover, we clearly demonstrate activity of ponatinib in a genetically defined colorectal tumor for the first time.

Finally, the choice of ponatinib as a RET inhibitor in clinical trials should be considered alongside the risk of ponatinib. Ponatinib has been associated with vascular occlusive events in clinical trials [11]. Thus, the appropriate monitoring of vascular health and a careful consideration of vascular comorbidities will help physicians decide when and where to use ponatinib in the clinical setting.

Collectively, these results provide strong support for the clinical evaluation of ponatinib in patients with RET fusion-positive cancers across 2 distinct pathological entities, NSCLC and CRC. These data were critical to the establishment of multiple clinical trials (including NCT01935336, NCT01813734, and NCT02272998).

## MATERIALS AND METHODS

### Ethics statement

Investigation has been conducted in accordance with the ethical standards and according to the Declaration of Helsinki and according to national and international guidelines and has been approved by the Crown Bio, ARIAD, and Champion’s Oncology institutional review boards.

### Reagents

Ba/F3 cell lines (DSMZ) were cultured as described previously [23]. Ba/F3 lines were cultured for less than 6 months (further cell line authentication was not conducted). Antibodies against RET, phospho-RET(Tyr^905^), extracellular signal-regulated kinase (ERK), phospho-ERK(Thr^202^/Tyr^204^), AKT, and phospho-AKT(Ser^473^) were obtained from Cell Signaling Technology (Danvers, MA, USA). Ponatinib was synthesized at ARIAD Pharmaceuticals and vandetanib, cabozantinib, sunitinib, sorafenib, and lenvatinib were obtained from a commercial vendor (Selleck Chemicals, Houston, TX, USA).

### Generation of engineered Ba/F3 stable cell lines

Ba/F3 cell lines were transformed to interleukin-3 (IL-3) independence by expressing constitutively activated versions of RET. *RET* cDNAs were synthesized in pLVX-IRES-Puro (Clontech Laboratories, Mountain View, CA, USA) by GenScript (Piscataway, NJ, USA) and Ba/F3 cells infected with lentiviral particles using the Trans-Lentiviral ORF Packaging Kit (Thermo Scientific, Waltham, MA, USA). Cells expressing RET were selected by IL-3 (R&D Systems, Minneapolis, MN, USA) withdrawal and puromycin (0.5-1 μg/mL, Invitrogen, Carlsbad, CA, USA).

### Viability assays

Cell lines were plated at densities that produced linear growth, treated with 8 concentrations of drug and viability assessed using CellTiter-96 AQueous One (Promega, Madison, WI, USA) after 72 hours. Data were plotted as percent viability relative to vehicle-treated cells and IC_50_s calculated using XLfit (ID Business Solutions, Guildford, UK).

### Immunoblotting

Cells were treated with ponatinib or vehicle for 1 hour. One hundred and twenty micrograms of clarified protein lysates (sodium dodecyl sulfate buffer) were subjected to western blotting using the indicated primary antibodies, horseradish peroxidase-conjugated secondary antibodies (Cell Signaling Technology) and the signal visualized with SuperSignal West Femto Substrate (Thermo Scientific). The pattern of bands produced is phospho-RET blots can be explained by an examination of the literature and the size of the different fusion proteins. Beyond the simple sizing of phospho-RET, the protein can show up as a single band or a double band. KIF5B-RET fusions tend to run as a single band while CCDC6-RET tends to run as a double band. Moreover across blots on different days in the same cell line, some have observed single versus double bands. [14, 22, 24]. The estimated size of the different RET fusion proteins are; KIF5B-RET 107kDa, CCDC6-RET 55kDa, NCOA4-RET 67 kDa. Please note that the primary PDX blots required long exposures, and that the bright bands are imperfections that are exaggerated upon long exposure.

### Engineered Ba/F3 *in vivo* models

All animal experiments were carried out under a protocol approved by the Institutional Animal Care and Use Committee. Tumors were established by subcutaneous implantation of engineered Ba/F3 cells into C.B-17 SCID mice (Charles River Laboratories, Wilmington, MA, USA). For efficacy studies, mice were randomized to treatment groups when the average tumor volume reached∼200 mm^3^. Mice were treated once daily by oral gavage with compound or vehicle (25 mM citrate buffer). The mean tumor volume of the treatment group was divided by that of the control group (at final measurement) to calculate percent tumor growth inhibition and tumor regression. For pharmacodynamic studies, tumor-bearing mice were treated with a single dose of compound for 6 hours. Tumors were harvested and protein lysates prepared in PhosphoSafe (Novagen, Madison, WI, USA) for western blotting.

### *In vivo* PDX studies

Efficacy studies using low passage PDX models representing human NSCLC (CTG-0170, CTG-0838, and CTG-1048) were performed at Champion’s Oncology (Baltimore, MD, USA) using female nu/nu mice. Studies representing human CRC (CR2518, CR1520, CR2502, and CR2520) were performed at Crown Biosciences (Beijing, P.R. China) using BALB/c nude mice.

### Assessment of tolerability

Instead of examining individual PDX models, we compared body weight changes across all PDX models. To do this we compiled all body weight measurements for all days for all models. Data were considered paired if they were taken at the same time point in the same model. To compare overall tolerability, we compared these percent body weight changes between ponatinib and chemotherapy using the Wilcoxon matched pairs signed rank test.

### RNA-seq PDX

To remove mouse tissue pollution reads in the raw RNA-seq reads, we mapped the raw reads to the human and mouse genome and transcript sequence, and identified the read origin by comparing its mapping condition. After filtering the mouse pollution reads, the gene expression was profiled using MMSEQ software. The gene fusion detection in Crown Bioscience’s PDXs was performed using the SOAPfuse, DeFuse, and TopHat-Fusion software. The GATK software was used to detect Gene SNP and Indel mutation.

### Fusion gene identification

Fusion genes in the PDXs of Champions Oncology (Hackensack, NJ, USA) were identified using the FusionSCOUT bioinformatics pipeline of MediSapiens (Helsinki, Finland) [25]. Briefly, paired-end RNA-seq data were aligned against the human genome GRCh37 and the Ensembl database v75 transcriptome definition. Fusion genes were identified based on discordantly mapping read pairs, followed by identification of exact fusion junctions based on junction spanning reads. Figures 2 and 4 were generated by aligning reads against the human genome using STAR aligner [26], with plots drawn using R Bioconductor package Gviz [27].

## SUPPLEMENTARY MATERIALS FIGURES AND TABLE



## Abbreviations

5-FU: 5-fluorouracil
ALK: anaplastic lymphoma kinase
ANOVA: analysis of variance
CRC: colorectal cancer
ERK: extracellular signal-regulated kinase
IC_50_: the half-maximum inhibitory concentration
IL-3: interleukin-3
i.p.: intraperitoneal injection
NSCLC: non-small cell lung cancer
PDX: patient-derived xenograft
q.d.: every day
RET: rearranged during transfection
SEM: standard error of the mean
TKI: tyrosine kinase inhibitor
