# Supplementary material for: RET fusions observed in lung and colorectal cancers are sensitive to ponatinib
Source: Oncotarget. 2018 Jul 3;9(51):29654–64. doi: 10.18632/oncotarget.25664 (PMC6049858; doi:10.18632/oncotarget.25664)
Supplement: Supplementary file 1 [file oncotarget-09-29654-s001.pdf]

RET fusions observed in lung and colorectal cancers are sensitive to ponatinib

SUPPLEMENTARY MATERIALS

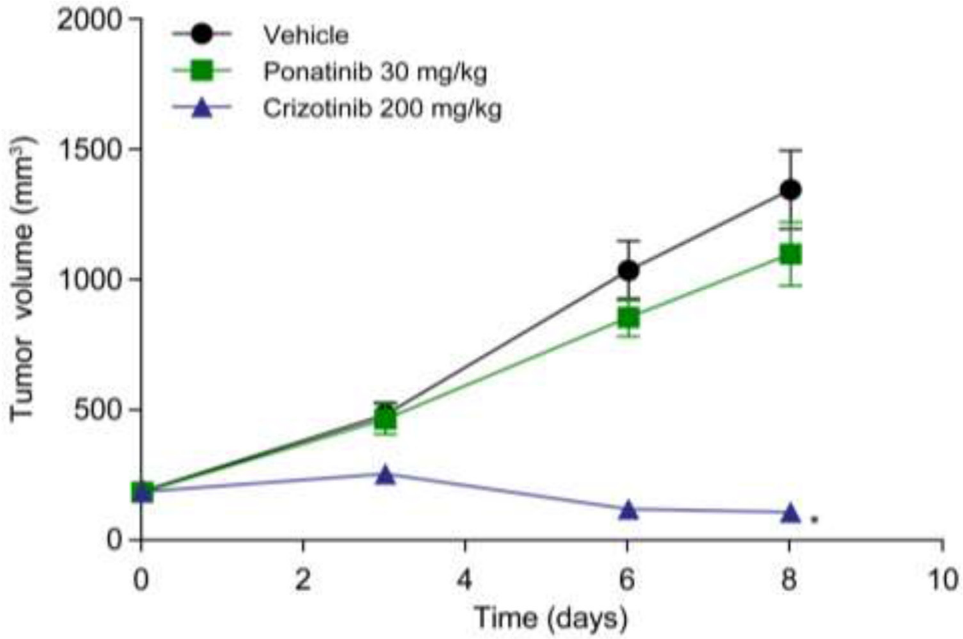

Supplementary Figure 1: Evaluation of ponatinib's selectivity in an isogenic Ba/F3 tumor model harboring EML4-ALK. Tumor-bearing animals were treated (by oral gavage) once-daily with vehicle or the indicated dose of either ponatinib or crizotinib for 8 days. The vehicle for ponatinib (citrate buffer) is shown, and nearly identical tumor growth was observed for the vehicle used for crizotinib (water; data not shown). Mean tumor volume and SEM are plotted. Each treatment group was compared with the relevant vehicle group using 1-way ANOVA ($^*P < 0.05$).

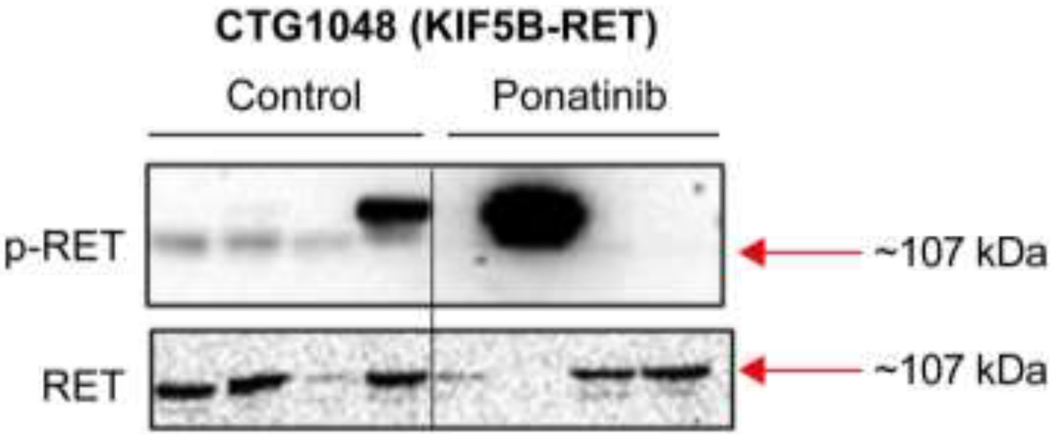

Supplementary Figure 2: Association of Ponatinib and p-RET Inhibition in KIF5B-RET-positive NSCLC PDX model, CTG-1048. Pharmacodynamic effect of ponatinib treatment in KIF5B-RET NSCLC PDX model CTG-1048, was assessed. Mice were administered a single oral dose of vehicle or ponatinib (20 mg/kg) and tumors were collected 6 hours later. Each lane represents a separate animal.

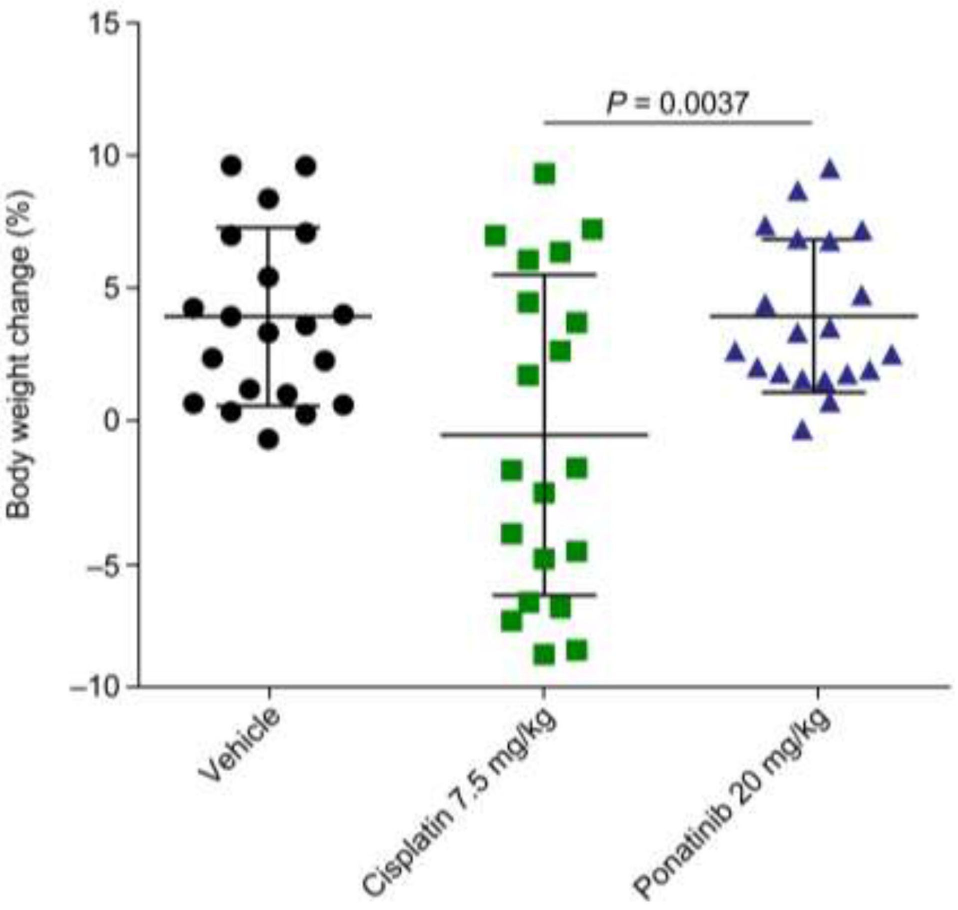

Supplementary Figure 3: The tolerability of ponatinib versus cisplatin. Ponatinib (20 mg/kg q.d. orally) and cisplatin (7.5 mg/kg once-weekly i.p.) were administered for 28 daysin 3 different mouse models. All body weight percent changes across all days for which paired data existed were compiled (see Materials and Methods).

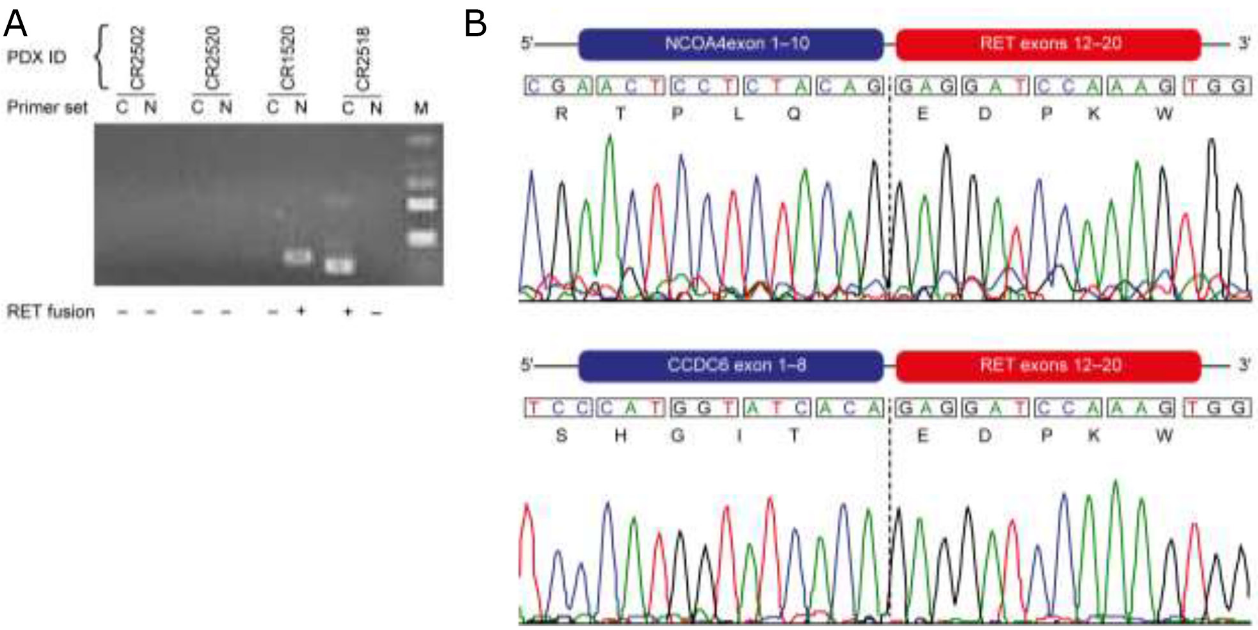

Supplementary Figure 4: Validation of the 2 CRC fusion gene breakpoint locations. **(A)** Detection of RET fusions by RT-PCR of RNA isolated from PDX tumor samples. C, CCDC6-RET primers; N, NCOA4-RET primers; M, Marker. **(B)** Validation of fusion gene breakpoint using Sanger sequencing of the cDNA.

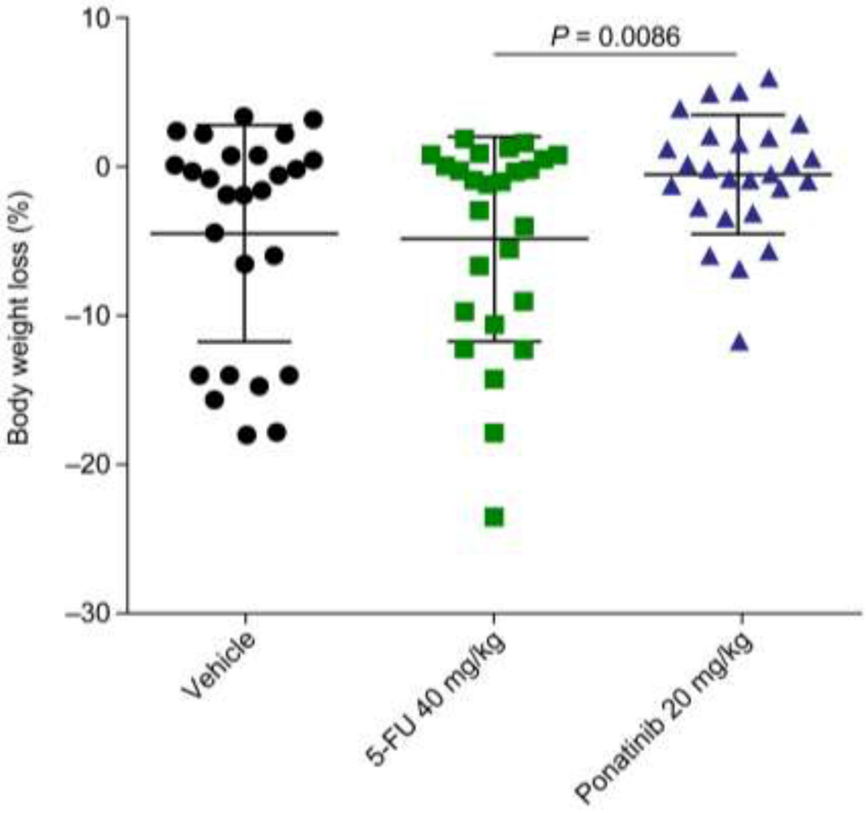

Supplementary Figure 5: The tolerability of ponatinib versus 5-FU. Ponatinib (20 mg/kg q.d. orally) and 5-FU (40 mg/kg daily) were administered for 28 daysin 4 different mouse models. All body weight percent changes across all days for which paired data existed were compiled.

Supplementary Table 1: Antitumor activity of ponatinib against NSCLC or CRC PDX tumor models *in vivo*

			Ponatinib	5-FU	Cisplatin	
**Model ID**	**Cancer type**	**RET fusion status**	**Tumor growth inhibition, %**	**Tumor regression, %**	**Tumor growth inhibition, %**	**Tumor growth inhibition, %**
CR2518	CRC	CCDC6-RET	-	78	0	-
CR1520	CRC	NCOA4-RET	79	-	62	-
CR2502	CRC	WT	41	-	68	-
CR2520	CRC	WT	24	-	72	-
CTG0838	NSCLC	KIF5B-RET	80	-	-	26
CTG1048	NSCLC	KIF5B-RET	-	11	-	95
CTG0170	NSCLC	WT	51	-	-	58

5-FU, 5-fluorouracil; CRC, colorectal cancer; NSCLC, non-small cell lung cancer; PDX, patient-derived xenograft; WT, wild type.
